# Case Report: Multi-modal motion-assisted memory desensitization and reconsolidation for traumatic grief (3MDR-TG)

**DOI:** 10.3389/fpsyg.2025.1548387

**Published:** 2025-09-30

**Authors:** Sophie M. C. Hengst, Mirjam J. Nijdam, Rebecca Gasser, Geert E. Smid

**Affiliations:** ^1^ARQ Centrum’45/ARQ National Psychotrauma Center, Diemen, Netherlands; ^2^Department of Psychiatry, University of Amsterdam, Amsterdam University Medical Centers, Amsterdam, Netherlands; ^3^University of Humanistic Studies, Utrecht, Netherlands

**Keywords:** traumatic, grief, 3MDR, multi-modal, prolonged grief disorder

## Abstract

**Background:**

The loss of a loved one under traumatic circumstances puts bereaved individuals at risk of developing prolonged grief disorder (PGD), (symptoms of) posttraumatic stress disorder (PTSD) and major depressive disorder (MDD), also referred to as traumatic grief. Traumatic grief is often associated with high symptom levels and strong avoidance. Not all patients benefit from standard treatments. Multi-modal Motion-assisted Memory Desensitization and Reconsolidation (3MDR) has shown to be effective in the treatment of treatment-resistant PTSD. The development of a 3MDR protocol specifically for Traumatic Grief protocol (3MDR-TG) enables grief-focused exposure in an immersive, personalized and activating context.

**Objective:**

To provide a proof-of-concept of 3MDR-TG for traumatically bereaved individuals with PGD and PTSD.

**Method:**

3MDR-TG was applied to a bereaved mother after the traumatic loss of her daughter. Qualitative interviews with the patient and the therapists were conducted to determine feasibility and acceptability. Clinician-rated PTSD, PGD and MDD symptoms were assessed at baseline, after 3MDR-TG, and at 16-week follow-up (primary endpoint).

**Results:**

The patient and therapists experienced the treatment as feasible and acceptable and rated the credibility of the treatment as high. Although some symptoms temporarily increased following exposure sessions, the overall decrease in PTSD, PGD and depressive symptom severity from baseline to primary endpoint (16 weeks follow-up) corresponded with reliable changes for PGD (RCI = −2.74), PTSD (RCI = −3.05) and depression (RCI = −3.05).

**Conclusion:**

We observed a clinically relevant and reliable decrease in PTSD, PGD and depressive symptoms over the course of 3MDR-TG. Further studies of 3MDR-TG in patients with traumatic grief are warranted.

## Introduction

Prolonged Grief Disorder (PGD) has only recently been included in the DSM-5-TR (American Psychiatric Association, 2022) and in ICD-11 (World Health Organization, 2019) as a formal bereavement-related diagnosis. PGD is characterized by core symptoms (intense yearning/longing for the deceased person and a preoccupation with thoughts or memories of the deceased person), accompanied by additional symptoms, e.g., intense loneliness, intense emotional pain (such as anger, guilt, and sadness), difficulty accepting the death, identity disruption, avoidance of reminders that the person is dead, feeling that life is meaningless, difficulty moving on with life and inability to experience a positive mood. These symptoms cause significant impairment in daily functioning that exceeds expected social, cultural, and religious norms. While both ICD-11 and DSM-5-TR have adopted the term PGD, there are differences between some of the diagnostic criteria: most importantly the time criterion (6 vs. 12 months post-loss in adults, respectively) and the minimum number of criteria required for a diagnosis (10 vs. 12, respectively).

PGD occurs in a significant minority of bereaved persons. After being confronted with natural loss, 3.4–9.8% develops PGD (Lundorff et al., 2017; Treml et al., 2022). Following violent or unnatural loss, the prevalence of probable PGD may be as high as 49%, according to a meta-analysis (Djelantik et al., 2020b). Emerging evidence shows that PGD tends to be more severe in the presence of comorbid PTSD symptoms (Djelantik et al., 2018; Heeke et al., 2017; Wen et al., 2022). PTSD can be diagnosed after direct or indirect exposure to a (traumatic) loss (Criterion A) and its symptoms are categorized by intrusive symptoms, avoidance of stimuli, negative alterations in cognitions and mood and alterations in arousal and reactivity for a minimum of a month (American Psychiatric Association, 2022).

The prevalence of comorbid PGD and PTSD in bereaved individuals has varied across studies and circumstances of the loss, e.g., 21% following a disaster (Kristensen et al., 2015), 16% following war and forced migration (Nickerson et al., 2014), and 15% following homicide (Rheingold and Williams, 2015). Meta-analyses show that PGD often co-occurs with other mental health problems, specifically, depressive, anxiety, and PTSD symptoms (Heeke et al., 2019; Komischke-Konnerup et al., 2021). These findings suggest that similar mechanisms may be present in individuals suffering from both PGD and PTSD. Both bereavement and trauma may luxate ‘traumatic distress’ (feelings of shock, anger, alertness, disbelief, detachment, loss of control and loss of faith in the world). A common response to traumatic distress is a tendency to anxiously avoid reminders of the traumatic event. ‘Separation distress’, is, other than traumatic distress, a feature specifically assigned to grief, described as a preoccupation with thoughts of the deceased, longing, searching behavior and proximity seeking (Prigerson et al., 1999). Separation distress gives rise to a different form of avoidance: avoidance of the *reality* of the loss and avoidance of a future (Boelen et al., 2006). Yet, both conditions can impact cognitions about oneself and ones identity, the future and their attachment to others.

Overlap in symptomology in PTSD and PGD may provide possibilities for treatment of this comorbidity. However, international guidelines for PTSD do not provide any recommendations for comorbid PGD or symptoms following traumatic loss. Evidence-based interventions for PTSD, such as prolonged exposure (PE), cognitive processing therapy (CPT), trauma-focused CBT, Eye Movement Desensitization and Reprocessing (EMDR), Narrative Exposure Therapy (NET) or Brief Eclectic Psychotherapy for PTSD (BEPP) (Hamblen et al., 2019) solely target posttraumatic symptoms and not grief. Cognitive behavioral therapy (CBT) has consistently found to be effective in the treatment of PGD, but studies have not systematically addressed effects of CBT for PGD on PTSD (Komischke-Konnerup et al., 2024; Bryant et al., 2024; Rosner et al., 2025).

Recently, various smaller and mostly uncontrolled studies have been compared by Eddinger et al. (2021), who evaluated the effects of different interventions for either PTSD or PGD on symptoms of comorbid PGD and PTSD (cognitive behavioral therapy, mindfulness, behavioral activation, online interventions, writing assignments, restorative retelling, EMDR, two-chair technique and other, less specified exposure based approaches). Of the 10 studies applying exposure based interventions, 7 showed an effect on both PGD and PTSD, although the effect on PTSD was more pronounced and more persistent at follow-up, e.g., after EMDR (*d =* 1.52 on PTSD and 0.28 on PGD; Sprang, 2001) and after imaginal exposure (*d* = 1.72 on PTSD and 0.42 on PGD; Asukai et al., 2011). In the two studies that evaluated an exposure-based treatment approach against a non exposure-based treatment, however, the effects on PTSD (0.79–0.99) and PGD (0.72–0.91) were much more comparable (Bolton et al., 2014; Eisma et al., 2015). Remarkably, behavioral activation showed the largest effect sizes for both PTSD and PGD (*d* = 2.45, Papa et al., 2013). These findings suggest that when treating PTSD and PGD integrally, both exposure and behavioral activation should not be left out and may be most efficacious.

Further investigation of the potential of existing interventions for PTSD in treating comorbid PGD and PTSD is ongoing, aiming to target overlapping symptoms such as avoidance and integration of the traumatic event in one’s life. Brief Eclectic Psychotherapy for Prolonged and Traumatic Grief (BEPPTG) (Smid et al., 2015) has been designed to combine various evidence-based interventions for PTSD, PGD, and depression, specifically Cognitive-Behavioral Therapy for Complicated Grief (Boelen et al., 2006) and Brief Eclectic Psychotherapy for PTSD (Gersons et al., 2015). In a cohort of refugees receiving BEPPTG, reductions in PGD and PTSD symptoms were strongly correlated: r(49) = 0.60, *p* < 0.01 (Djelantik et al., 2020a). However, in this study, BEPPTG was embedded in a day treatment program with several other supportive interventions. The effects on PTSD and PGD were medium-sized (d = 0.61 on PGD, 0.34 on PTSD). Notably, modest effects of PTSD treatments in refugees compared with other populations have consistently been reported (e.g., ter Heide and Smid, 2015). Indeed, a recent meta-analysis found no clear differences in the efficacy of psychosocial interventions compared to treatment as usual for reducing symptoms of PTSD, depression, and anxiety in refugees (Turrini et al., 2025). Imaginary Rescripting (ImRs) (Kip et al., 2023) is an example of an effective and generally well-tolerated intervention for PTSD, but is still studied as possible intervention for PGD (Lechner-Meichsner et al., 2024).

A strategy to augment effects of psychotherapeutic interventions is virtual reality (VR). VR has shown to be at least equally effective for PTSD as regular treatment (Eshuis et al., 2021; Gonçalves et al., 2012). Several VR-interventions for PGD have been developed, though carefully studied and debated: creating a hologram or ‘illusion’ of the dead being alive may not at all be helpful in facing the reality of the death (Pizzoli et al., 2023). Adding a digital landscape and photo material to conventional CBT has shown beneficial effects on symptoms compared to CBT alone (Quero et al., 2019) in a small sample of patients with adjustment disorder and PGD at follow-up after 12 months.

An innovative VR-treatment, combining a virtual environment, pictures, exposure and activation by motion is Multimodal Motion-assisted Memory Desensitization and Reconsolidation (3MDR). 3MDR has been developed in Netherlands and has been applied to increasing numbers of treatment-resistant veterans with PTSD (Bisson and Van deursen, 2020; Hamilton et al., 2021; Jones et al., 2022; Van Gelderen et al., 2020). The protocol for PTSD involves 6 sessions of exposure to personally selected pictures and music associated with the traumatic events, projected on a 180 degrees screen in a virtual reality environment whilst walking on a treadmill. Recently published RCTs in veterans with treatment-resistant PTSD and in those with mild traumatic brain injury contributed to the evidence base regarding 3MDR’s effectiveness on PTSD, depression and anxiety (Bisson and Van deursen, 2020; Roy et al., 2022; Van Gelderen et al., 2020).

So far, 3MDR has not been applied in patients with PGD and PTSD. However, 3MDR can flexibly integrate different interventions for PGD: facilitating access to memories and emotions around the lost loved one (enhanced by the virtual environment and music), exposing the patient to the reality of the loss, minimizing anxious avoidance, exploring cognitions around the loss and the deceased, attribution of meaning in relation to one’ s self/identity and future and activation by walking on the treadmill. This resulted in a new protocol: 3MDR for Traumatic Grief (3MDR-TG) that combines grief- and trauma-focused exposure with simultaneous behavioral activation.

Aim of the current study was to describe the 3MDR-TG protocol, to report a first application of the protocol in a patient and to evaluate its feasibility and acceptability. Rationale and motivation for applying 3MDR-TG in this specific patient was the presence of PTSD and PGD symptoms with prominent avoidance symptoms following a traumatic loss following insufficient response to previous evidence-based PTSD treatment. We described the course of symptoms of traumatic grief (PTSD, PGD and depression) from pre-treatment to post-treatment and follow-up at 16 weeks. We hypothesized that the grief-related interventions comprising the new 3MDR-TG protocol, such as intense exposure to the traumatic aspects of the loss, exposure to the reality of the loss, reconsolidation of the memory of the loss and correction of dysfunctional cognitions and activation/future orientation, would be feasible and acceptable for the patient as well as for the therapists.

## Method

### Setting

The participant was recruited from ARQ Centrum’45, a Dutch mental health institute specialized in the treatment of psychopathology associated with trauma and loss. Following a consultation by the medical ethics committee of University Medical Centre Utrecht in Netherlands (file number 21-372/H-C), the study was exempted from formal review. Written informed consent was granted by the patient to undergo the innovative treatment and to publish this case report, anonymized by using pseudonyms.

Two therapists were involved in the treatment and present during all sessions, dividing the tasks (operator of the installation vs. therapeutic interventions). Both therapists had >5 years of experience in the application of different psychotrauma and grief treatments.

In case of increasing distress during treatment, the patient could contact the treatment team during office hours.

3MDR is a manualized intervention, developed through collaboration of the Military Rehabilitation Center, Military Mental Health Care, and Motek Medical B. V. (all located in Netherlands), and was used as described in the protocol (Van Gelderen et al., 2018).

### Description of the 3MDR-TG protocol

The 3MDR-TG protocol is an adaption of the 3MDR protocol for PTSD, developed by Vermetten et al. (2013, 2025). Whilst 3MDR consists of 6 weekly sessions, consistently focusing on seven traumatic memories or aspects thereof, 3MDR-TG consists of 10 weekly sessions with 4 different phases. During the first two sessions, psycho-education on traumatic grief and an explanation of the 3MDR-TG sessions are provided, grief-related avoidance is explored, and pictures and music related to the loss are selected (*preparation* phase). The 8 remaining sessions each last 90 min and take place in a virtual reality environment and treadmill. They involve the *exposure phase* (3 sessions), *revising cognitions and behavior phase* (1 session + 2 optional sessions) and *identity and future phase* (2 sessions). Every treadmill session starts with a brief evaluation of last week, followed by a platform phase (walking continuously on a treadmill with safety harness towards a 180 degrees projection screen, while guided by a therapist) and a post-platform phase (debriefing). During the platform phase, the patient first performs a warming up (3–4 min) while the virtual reality environment displays a neutral landscape and plays the selected music that reminds the patient of the deceased loved one. After the warming up, the virtual environment changes: the patient walks into a tunnel, where at the end an image appears. The image gradually increases in size during walking, as if coming nearer.

The *exposure* to the full-screen image lasts several minutes, while the therapist asks the patient to describe the image and the memory that is connected to the image. The therapist then asks which emotions and physical sensations come up at that moment. The therapist types the patients’ words so that they appear on the image. An oscillating red ball then appears on the screen, presenting numbers on it from 0 to 99. The patient is asked to follow the red ball with the eyes and read the numbers out load as they appear on the ball. After 30 s of following the red ball, the image disappears. After the last image, another piece of self-selected music is played for a cool-down period, helping the patient reconnect to the present.

For each of the three sessions of exposure (sessions 3–5), 6 to 7 images associated with the traumatic loss are selected, mutually approved by therapist and patient. During the first two exposure sessions, exposure to the (traumatic) circumstances of the death is carried out, whilst the third session involves exposure on the relationship with (and positive memories of) the loved one.

During the *revising cognitions and behavior phase* (sessions 6–8), several other grief interventions are integrated into the protocol: session 6 encompasses a virtual visit to the grave to facilitate an imaginary conversation with the deceased. Session 7 is reserved for virtual exposure to specific situations, stimuli or places associated with the loss that are avoided; session 8 is reserved for revising excessive grieving behavior (both session 7 and 8 are optional and applied if avoidance and excessive grieving are strongly present). During the *identity and future phase* (sessions 9–10), the patient is asked to select images that reflect one’ s own identity, symbols of qualities, ambitions, wishes, dreams, for now and the future. For perusal, the protocol can be retrieved through the authors (Hengst and Smid, 2022).

### Measures

A thorough assessment by an independent assessor took place before starting treatment (T_0_), after finishing treatment (T_1_) and 3 months after T_1_ (follow-up, T_2_). The assessments included administration of the Dutch version of the Clinician-Administered PTSD Scale for DSM-5 (CAPS-5) (Weathers et al., 2018; Boeschoten et al., 2018), The CAPS-5 consists of 20 items measuring PTSD symptoms in 4 symptom clusters (intrusions, avoidance, alterations in mood/cognitions and hyperarousal). Questions are rated on a 5-point scale, with the total symptom severity score ranging from 0 to 80. Higher scores reflect more severe PTSD. The Dutch translation (Boeschoten et al., 2018) shows high internal consistency and interrater-reliability (Cronbach’s alpha = 0.90, ICC = 0.98).

The Traumatic Grief Inventory-Clinician Administered (TGI-CA) is an interview that is developed to assess symptom severity of PGD and to diagnose PGD according to criteria in DSM-5-TR and ICD-11. The 22-item TGI-CA is based on the 22-item Traumatic Grief Inventory-Self Report Plus (TGI-SR+, Boelen et al., 2018; Boelen and Smid, 2017). The English and Dutch translation of the TGI-CA are freely available *via* the Open Science Framework (https://osf.io/a6hmc/). The TGI-SR + is a reliable and valid survey to assess PGD symptoms in terms of ICD-11 and DSM-5-TR (Lenferink et al., 2022, 2023).

The Dutch version of the Hamilton Depression Rating Scale (HDRS) (Hamilton, 1960; de Jonghe, 1994) is a standardized tool used by clinicians to assess the severity of depressive symptoms and monitor treatment progress. It is a questionnaire with 17 items, where clinicians rate a patient’s symptoms based on a structured interview. The total score is calculated by summing the individual item scores, and higher scores indicate more severe depression. The translation in Dutch (de Jonghe, 1994) has shown good validity and high reliability (Kupka et al., 1996). Total scores of the CAPS-5, TGI-CA and HDRS were used to measure the severity of symptoms of PTSD, PGD, and depression, respectively.

Weekly assessments from start to end of treatment involved the Brief Traumatic Grief Questionnaire (BTGQ; Djelantik et al., 2016), a 12-item self-report questionnaire specifically developed for this study (Supplementary material 1 provides the full BTGQ). Four symptoms of PGD, four symptoms of PTSD, three symptoms of depressive disorder (depressed mood, lack of interest, feelings of guilt) and a question on level of functioning were rated on a scale from 0 (never) tot 10 (always) to measure the response to every session.

Feasibility and acceptability of the treatment was evaluated with the patient at T_1_ and T_2_, in a qualitative interview. Both therapists were asked to share their experience on different aspects of working with the 3MDR-TG protocol and the 3MDR installation as well.

### Statistical analysis

The Reliable Change Index was used to establish whether differences between total scores obtained at T_0_, T_1,_ and T_2_ were primarily due to treatment rather than measurement error. The RCI was calculated according to the method outlined by Jacobson and Truax (1991), with SD based on earlier validity studies of the TGI-SR (SD = 12–14, Lenferink et al., 2022), CAPS-5 (SD = 14) (Boeschoten et al., 2018), and HDRS (SD = 6–8) (Bobo et al., 2016).

## Case description

### Patient

The patient, Robin (a pseudonym) was a 46-year-old Native Dutch woman, studying to be a counsellor. She was living together with her partner and two sons (15 and 13 years old), after losing her youngest child, a daughter Cato, 5 years ago. Cato had suffered from a congenital disorder from birth up to her death at the age of 5. The 5 years of care for Cato included 65 hospitalizations due to chronic feeding problems, underlying lung and heart conditions and common epileptic insults, after which she died due to acute bowel obstruction. Months after Cato’s death, Robin had received EMDR treatment with no effect. She sought treatment again 4 years later because of increasing exhaustion. During assessment at our center, she reported intrusions of hospitalizations of her daughter, anxiety for losing her sons (due to illness or harm), avoidance of specific photographs of her daughter, avoidance of more general triggers (hospitals, ambulances, needles, plasters) which conflicted with some of the situations she encountered during her work at the hospice. She reported agitation, concentration problems, forgetfulness, severe exhaustion and feelings of guilt. There were negative cognitions about herself, about the future and a feeling of disconnection from her loved ones.

Before the start of the treatment (at T_0_), Robin’s CAPS-5 score was 29, meeting all criteria for the PTSD diagnosis. Her score on the TGI-CA was 52 (Lenferink et al., 2022), meeting all criteria of PGD conform DSM-5-TR (American Psychiatric Association, 2022) and ICD-11 (World Health Organization, 2019). She did not fully meet criteria of a depressive disorder conform DSM-5-TR on the HDRS (score 16). She did not meet the criteria of any other mental disorder and had no history of mental disorders in the past. After further screening and assessment with independent psychologists, Robin was diagnosed with PTSD and PGD according to DSM-5-TR (American Psychiatric Association, 2022). Robin did not receive any other psychological or pharmacological treatment over the course of the study.

### Clinical description of the therapy sessions

#### Phase 1: preparation

Session 1 involved psycho-education on symptoms of traumatic grief and identification of the foremost avoided triggers reminding Robin of her daughter’s illness and death. The therapist asked Robin to select 14 images related to Cato’s illness and death for the first two exposure sessions (e.g., inside an ambulance, daily medicine routine, the medicine, hospital bag, Cato being ill, Cato’s arm in a drip and her belly plastered). Additionally, Robin selected one song that would bring her back to the period of Cato’s death and soothing nature sounds to reconnect with the present.

During session 2, the selection of pictures was discussed and Robin was familiarized with the virtual environment and the protocol.

#### Phase 2: exposure

Session 3 was the first session on the treadmill. Exposure to seven images took place, feeling angry, lost, and powerless about what Cato had been through. The week after, she reported feeling exhausted and with a strong longing for Cato after seeing her so clearly on the screen.

Session 4, seven other pictures were used for exposure. Pictures related to Cato’ s medicine routine brought up a feeling of purpose about the care she offered, “for the first time in my life I did something I was seriously good at.” Photographs of Cato dying and just after passing brought up much emotional pain and guilt. Robin wondered if this treatment would help her or would worsen her symptoms instead. For the next session, Robin was asked to select 7 photo’s that reflected her relationship (positive memories) with her daughter.

Session 5 focused on memories of Robin and Cato together. Robin chose images from the period after Cato’s birth, up to 5 years of age. Robin expressed feelings of longing and missing, pride and sadness, missing the only female companion in the family.

#### Phase 3: revising cognitions and behavior

Session 6, Robin was invited to virtually walk towards a photograph of Cato’ s grave, describing the memories of the burial, the process of designing the grave and its symbols around it. She was invited to imagine being there and to say what she wanted to say to Cato. This brought up expressions of strong love, longing and missing, but also a sense of needing to let go, as “she knew this would be the last image of Cato.”

In preparation of the next session, remaining dysfunctional cognitions were explored, such as guilt, remorse, anger, ambivalence and regret. Robin reported a persistent anxiety around the wellbeing of her sons and feelings of guilt about not having been as available for them as she wanted. She selected 5 pictures of her sons.

Session 7, Robin was exposed to these pictures, which initiated an imaginary conversation between her and her sons. It brought up sadness, for them, their loss of a little sister, loss of their earlier ‘ungrieving’ mother and loss of faith. She allowed herself to express the fear she felt for their health and safety and apologized to them, for all of it.

Robin did not report excessive grieving behavior. Session 8 was therefore used (on request of the patient) for exposure to two photographs that she had avoided earlier in the phase of treatment: one of Cato’ s face when she was dying, and one of Cato being laid out after her death. Exposure to these two photo’s in session 8 brought up anger, an “unwillingness to let her pass away from this earth” and after that, pain, sadness and reality of the loss.

#### Phase 4: identity and future

Session 9 was planned for evaluating the symptoms and transferring the focus to the future. Despite a recent feeling of depression, exhaustion and acute grief after last session, Robin did however feel stronger than before and more aware of the different aspects of her pain. For the last session, Robin was asked to bring images that she associated with her close future to the last session.

Session 10 encompassed exposure to images of her holiday home in France, vegetable garden, running, an atelier and a diploma, which was experienced as empowering.

A final evaluation (session 11) took place with her partner.

## Results

Total scores of CAPS-5, TGI-CA and HDRS, assessed at baseline (T_0_), end of treatment (T_1_) and 16 weeks follow-up (T_2_) are shown in Table 1. TGI-CA total scores, measuring PGD symptom severity, decreased overall, with a strong decrease of symptoms between T_0_ and T_1_ and a slight increase of PGD symptoms at T_2_, which appeared to be related to the yearly date of death of her daughter, which was around the time of the last assessment. Overall, the difference in TGI-CA score measured at T_0_, T_1_ and T_2_ (RCI: −2.74) can be considered as a reliable change.

**Table 1:** Pre- and post-treatment and 4-month follow-up symptom levels of PTSD, PGD, and depression.

Questionnaire	Pre-treatment	Post-treatment	4-month follow up
HDRS	16	3	6
TGI-CA	52	26	34
CAPS-5	29	14	9

HDRS, Hamilton Depression Rating Scale; TGI-CA, Traumatic Grief Inventory-Clinician Administered; CAPS-5, Clinician-Administered PTSD Scale for DSM-5.

HDRS total scores, measuring depressive symptom severity, decreased between T_0_ and T_1_, with a slight increase of symptoms at T_2_, indicating a reliable change (RCI: −3.05).

**Figure 1:**
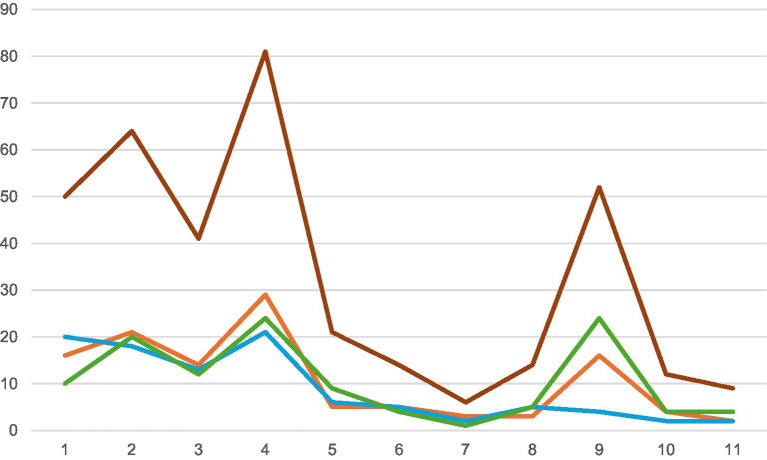
Total symptoms per session measured by the Brief Traumatic Grief Questionnaire (BTGQ, Djelantik et al., 2016) (sessions 1–11). The graph shows the BTGQ total scores based on 12 items (PTSD, PGD and depression), measured every session (total 11) reflecting on the effect of the session before. Y-axis: Scores BTGQ. X-axis: session numbers. Red: BTGQ total scores per session, blue: PTSD symptom levels per session, green: depressive symptoms per session, orange: PCRS symptoms per session.

CAPS-5 total scores, measuring PTSD symptom severity, decreased at T_1_, to further decrease at follow-up (T_2_), indicating a reliable change (RCI: −3.05). On different domains, improvement was visible (Figure 1).

In Figure 1, the pattern of response per session is visualized, based on the assessment by the BTGQ, showing the evolution of symptoms per session. Exposure to the traumatic circumstances and reality of the loss (sessions 3–4, session 9) exacerbated symptoms of PTSD, PGD and depression. These elevations were temporary and common for exposure-based therapies.

During the first evaluation at T_1_, patient mentioned having no more intrusions and experiencing no more tendency to avoid. She was able to access positive memories of her daughter, had more energy and less anxiety around her son’s safety. Her partner confirmed these improvements: the atmosphere at home had improved in the last month of treatment. There was more flexibility around how to remember Cato, especially on specific dates. There was less agitation, more energy and less estrangement. The evaluation of the treatment at T_2_ remained positive.

Both at T_1_ and T_2_, patient was interviewed regarding the feasibility and acceptability of the treatment. The patient having experienced the protocol as effective and helpful. “*3MDR really made me undergo everything and give me the feeling that I could not avoid my emotions any longer.” “I now understand better why Cato is such a part of my identity and what it is that I miss so much. I also know better who I am without her*.” Working with photo’ s, music and physical activity (walking) was evaluated as an additional and beneficial effect. The element of walking enhanced the feeling of “*having worked hard on my grief*,” although she mentioned often being exhausted after a session after walking for at least 1 h, needing to recover for several hours at home. However, this investment was acceptable for the patient. The temporary exacerbation of symptoms, usually 24–48 h after sessions, was comprehensible and bearable for the patient, and besides explanation needed no extra interventions. The notion of a clear start and end point additionally helped her to accept and commit to the treatment period.

Both therapists and patient experienced the application of the protocol as feasible: the composition of the sessions and the amount of sessions (11 in this patient) was appropriate. The calculated time per session (90 min) proved to be the minimum amount of time needed to work on 7 photographs. Preparation (30 min per session) for the therapists was more than treatment as usual but feasible. Working with the 3MDR installation was uncomplicated, although one session needed to be cancelled because of technical problems with the screen.

## Discussion

This case study presents a first description of Multi-modal Motion assisted Memory Desensitization and Reconsolidation for Traumatic Grief (3MDR – TG), applied to a patient suffering from PTSD and PGD after the loss of a child. The 3MDR-TG treatment protocol integrates multiple interventions known to be effective in treating PGD, PTSD and depressive disorder: (1) exposure to memories and triggers that relate to the (traumatic) circumstances of the death, whilst (2) walking, which involves approaching the previously avoided traumatic memories, (3) applying a dual-attention task to facilitate desensitization and reconsolidation; (4) exposure to the reality of the death, enhanced by motion to facilitate acceptance of the loss; (5) exposure to the relationship with the deceased, to facilitate catharsis and explore forms of identity loss; (6) altering negative and dysfunctional cognitions, by facilitating a symbolic interaction with the deceased and (7) visualizing a future without the deceased and moving towards these images, creating positive appraisal and meaning-making. All the above aspects are enhanced by an active, side-by-side therapeutic setting, increasing treatment engagement and overcoming avoidance. The results of this proof-of-concept trial enable the generation of hypotheses about 3MDR-TG effects on PTSD and PGD following traumatic loss that will be discussed below.

In this study, the patient showed improvement on all of the above grief-related domains. The longing for the patients daughter had been very strong during the sessions, but became more dynamic in the end of treatment, allowing herself to talk and think about other things and finally integrating her daughter in her new life. This longing, which has been described by Pizolli as a possible negative effect on grief when using VR, had no negative effect on the process of the patient nor stagnating effect of treatment (Pizzoli et al., 2023). The overall improvement on prolonged grief symptoms is a hopeful finding and suggests that ‘separation distress’ (Prigerson et al., 1999), avoidance of the reality of the loss and avoidance of a future (Boelen et al., 2006) is addressed with this immersive treatment. The patient also reported improvement on posttraumatic stress symptoms, e.g., avoidance of internal and external triggers, intrusions and agitation, which was conform our expectations: earlier RCTs have shown effectiveness of 3MDR on PTSD symptoms (Van Gelderen et al., 2020).

The promising course of depressive symptoms in this patient is an additional benefit. This is in line with the effects found by Jones, reporting significant improvement on the PHQ-9 in 11 patients (Jones et al., 2022). This may partly be assigned to the activating aspect of 3MDR, as the patient spends an hour per session on the treadmill. As more studies have shown positive effect of activation on grief, walking as augmentation strategy within 3MDR treatment may have important additional benefits (Eddinger et al., 2021).

### Study strengths and limitations

The 3MDR-TG treatment protocol has been feasible and acceptable for the patient. Credibility of the treatment was rated as high by both patients and therapists. Targeting both separation distress symptoms as well as traumatic stress symptoms by multiple ways of exposure, combined with the facilitation of future orientation and activation, resulted in an improvement of symptoms of ‘traumatic grief’ in this patient. The decrease in PTSD and PGD symptom severity from baseline to end point in a clearly protocolized, limited treatment program (11 sessions) corresponded with a clinically relevant response. These therapeutic outcomes were maintained at follow-up. Post-treatment and at follow-up, the patient did not meet the diagnostic criteria of any DSM-5 diagnosis.

This study further illustrates the use of technologically supported multisensory cues in treatment of traumatic grief, which is still scarcely studied. In addition, this study shows how virtual reality and multisensory cues can be used to enhance various forms of grief- and trauma-focused exposure.

Limitations of this study are partly inherent to its design as a single-case study: lack of randomization and poor generalizability, leading to an outcome that is rather hypothesis-generating rather than conclusive (Lobo et al., 2017); no causal inferences can be made based on the data of a single patient. Different populations may respond differently to 3MDR depending on chronicity of their symptoms and their treatment history (Nijdam et al., 2023). The treatment history of the patient in this case report involved solely evidence based treatment for PTSD (EMDR) and no other grief-related treatment. It is unknown if the patient would have benefited from other evidence-based grief interventions.

Inherent in this treatment is the dependency on technology. While investigating 3MDR-TG and its indication, additional costs related to the purchase and use of the equipment need to be taken into account. If proven (cost-)effective in further studies, dissemination would depend on an infrastructure with a treadmill and other hardware components, and specific training. Adaptations with f.e. a portable VR-system may be investigated in further research as well, to enhance scalability. In addition, a basic level of mobility is required from the patient to be able to follow this treatment.

The temporary increase of symptoms during exposure, reported on the BTGQ, is commonly seen in exposure-based treatment options and often seen as ‘a temporary side-effect’ of exposure based treatment (generally 24–48 h after sessions). Patients need to be informed about this possibility, should be monitored and if needed, offered extra guidance.

### Clinical and research implications

The use of the 3MDR-TG protocol was found to be feasible and acceptable for both patient and therapists. Symptoms of PGD, PTSD and depression reliably improved in this patient. 3MDR-TG seems to offer a limited and comprised treatment, involving the most relevant aspects of traumatic grief.

Further research is warranted to replicate and extend these findings, using heterogenous and larger samples, particularly among patients who did not benefit from evidence-based treatments for PTSD and PGD. This may offer further insight into the relevance for 3MDR-TG in the field of traumatic grief treatment. Open-label feasibility trials and randomized controlled trials (comparing 3MDR-TG with other, evidence-based, interventions) are needed as next step to investigate efficacy.

## Conclusion

In this case study, a newly developed protocol for a virtual reality intervention with motion was described for traumatic grief, typically consisting of PGD, PTSD and MDD symptoms. The protocol was found to be feasible and acceptable to both patient and therapists. A clinically relevant decrease in PGD, PTSD and MDD symptoms was demonstrated over the course of 3MDR-TG in a single patient. These data are a hopeful starting point for further investigating 3MDR-TG in patients with PGD and PTSD, warranting further study.

## Data Availability

The raw data supporting the conclusions of this article will be made available by the authors, without undue reservation.
