# Supplementary material for: Case Report: Multi-modal motion-assisted memory desensitization and reconsolidation for traumatic grief (3MDR-TG)
Source: Front Psychol. 2025 Sep 30;16:1548387. doi: 10.3389/fpsyg.2025.1548387 (PMC12518244; doi:10.3389/fpsyg.2025.1548387)
Supplement: Supplementary file 1 [file Data_Sheet_1.docx]

**Supplement 1**

**The Brief Traumatic Grief Questionnaire**

This questionnaire is designed to quickly evaluate your complaints in the field of persistent complex grief, post traumatic stress and depression. The first part of the questionnaire will be completed by you, the second part by your practitioner.

Part 1

| **Complaints** | **This statement applied to me *last week*.**  The 0 means: does not apply to me at all. The 10 means: completely applicable to me |
| --- | --- |
| 1. I found myself longing or yearning for the person who died. | 0 – 1 – 2 – 3 – 4 – 5 – 6 – 7 – 8 – 9 – 10 |
| 1. I had trouble accepting the loss. | 0 – 1 – 2 – 3 – 4 – 5 – 6 – 7 – 8 – 9 – 10 |
| 1. I avoided places that reminded me that the person I lost has died. | 0 – 1 – 2 – 3 – 4 – 5 – 6 – 7 – 8 – 9 – 10 |
| 1. I experienced confusion about my role in life | 0 – 1 – 2 – 3 – 4 – 5 – 6 – 7 – 8 – 9 – 10 |
| 1. I had recurring memories or dreams about the events surrounding my loved one's death | 0 – 1 – 2 – 3 – 4 – 5 – 6 – 7 – 8 – 9 – 10 |
| 1. I avoided memories, thoughts, or activities of my loved one's death | 0 – 1 – 2 – 3 – 4 – 5 – 6 – 7 – 8 – 9 – 10 |
| 1. I felt like I have no future | 0 – 1 – 2 – 3 – 4 – 5 – 6 – 7 – 8 – 9 – 10 |
| 1. I became upset when I remembered the death of my loved one | 0 – 1 – 2 – 3 – 4 – 5 – 6 – 7 – 8 – 9 – 10 |
| 1. I was in a gloomy mood | 0 – 1 – 2 – 3 – 4 – 5 – 6 – 7 – 8 – 9 – 10 |
| 1. I had little interest in activities | 0 – 1 – 2 – 3 – 4 – 5 – 6 – 7 – 8 – 9 – 10 |
| 1. I felt guilty | 0 – 1 – 2 – 3 – 4 – 5 – 6 – 7 – 8 – 9 – 10 |
| 1. I had trouble functioning in everyday life | 0 – 1 – 2 – 3 – 4 – 5 – 6 – 7 – 8 – 9 – 10 |

English translation by the authors from the Dutch version of the BTGQ (Djelantik, Boelen, & Smid, 2016).
